# PET/MRI reveals ongoing metabolic activity in ACL grafts one year post-ACL reconstruction

**DOI:** 10.1186/s40634-020-00258-2

**Published:** 2020-06-01

**Authors:** Seth Korbin, Michael Salerno, Justice U. Achonu, Mingqian Huang, Paul Vaska, Amanda Pawlak, David E. Komatsu, James M. Paci

**Affiliations:** 1 Department of Orthopaedics, Stony Brook University Hospital, Stony Brook, New York, USA; 2 Department of Biomedical Engineering, Stony Brook University, Stony Brook, New York, USA; 3 Department of Radiology, Stony Brook University Hospital, Stony Brook, New York, USA; 4 Orlin & Cohen Orthopedic Group, Zucker School of Medicine at Hofstra/Northwell School of Medicine, Hempstead, New York, USA

**Keywords:** Anterior cruciate ligament, Positron-emission tomography, Magnetic resonance imaging, Autograft

## Abstract

**Purpose:**

To use serial PET/MRI imaging to radiographically evaluate the metabolic activity of the ACL graft over the first post-operative year.

**Methods:**

Six patients undergoing primary ACL reconstruction were recruited in this prospective study in an inpatient university hospital. All patients underwent femoral and tibial suspensory cortical fixation with quadrupled semitendinosus autograft hamstring ACL reconstruction by an orthopaedic surgeon. Simultaneous ^18^F-FDG PET and MRI of both the operative and non-operative knee was performed at three, six, and 12 months post-operatively. Quantification of the mean standardized uptake value (SUV) within the whole-knee, as well as tibial tunnel, femoral tunnel, and intra-articular graft regions of interest (ROIs).

**Results:**

PET whole-knee activity was increased at all time-points post-operatively compared to the control, non-operative knee. Activity decreased over time, yet considerable generalized activity remained 1 year post-operatively, with relative intensity 34% percent higher than control. When the operative knee was divided into three whole-regions, there was greater activity in the tibia at three than 12 months, the femur at six than 12 months, and in the tibia compared to the intra-articular region at 3 months. When they were separated into sub-regions, results demonstrated greater activity closer to the joint surface.

**Conclusions:**

PET/MRI evaluation of ACL graft reconstructions demonstrates evolving biologic activity within the graft and both tunnels. Focal areas of increased activity within the tunnels may indicate of ligamento-osseous morphologic changes. These data suggest that graft incorporation continues well beyond 1 year post-operatively.

**Level of evidence:**

Level IV.

## Introduction

The current standard of care for anterior cruciate ligament (ACL) injuries involves the use of quadriceps, bone-patellar tendon-bone, hamstring autograft, or allograft tissues. The biologic incorporation of these grafts has been well-studied using histologic evaluation, animal models, and radiographic assessment. Studies suggest that the tendon graft initially undergoes a process of peripheral synovial vascularization and ultimately achieves ultrastructural incorporation [21, 24]. Post-operative magnetic resonance imaging (MRI) scans of ACL grafts support this notion [16]. However, information is lacking regarding the true biologic and metabolic activity of ACL tendon grafts during the post-operative period.

Another imaging modality, SPECT/CT (Single-photon emission computed tomography/computed tomography), has previously been used with diphosphonate bone tracers to evaluate changes in biomechanical loading and the subchondral bone plate [12]. Higher amounts of this bone tracer uptake has been shown to be significantly related to a higher degree of osteoarthritis in knees post- ACL reconstruction [14]. While this imaging modality serves to measure additional parameters (in vivo joint loading, bone tunnel remodeling and graft incorporation), interpretation is required to differentiate between an inflammatory response versus altered in vivo loading or both [13]. MRI is the gold standard for assessing morphological and structural changes in patients post-ACL reconstruction [17]. Therefore, we chose to combine positron emission tomography (PET), an effective tool for evaluating metabolic activity, with MRI, to demonstrate a more accurate picture of the biological activity within the knee.

^18^F-FDG PET is a functional imaging technique that uses a radioactive analog of glucose to assess glucose metabolism in body tissues. In addition to its well-established role in oncologic and brain imaging, ^18^F-FDG PET is also used in the assessment of infection, inflammation, and more recently, tissue repair [7, 10, 19, 23, 25]. ^18^F-FDG accumulates in regions proportionally to their glycolytic rate, therefore greater uptake is seen in regions of increased metabolic activity. In orthopaedic applications, ^18^F-FDG-PET has been utilized in the management of patients after arthroplasty and has been effective in evaluating a variety of infectious and inflammatory processes [25, 26].

The feasibility of combined PET/MRI imaging to assess ACL graft healing following surgery has also been investigated, although current research is exploratory. PET has been utilized in combination with MRI to assess FDG uptake of the knee in a canine model of anterior cruciate ligament transection (ACLT) [15]. In this study, five skeletally mature beagles were imaged with PET/CT and MRI pre-operatively and 3, 6, and 12 weeks after ACLT, with the contralateral knee serving as the control. The technique was sensitive to metabolic changes in different structures of the knee joint, suggesting the potential use of FDG as a biomarker for graft viability. In a recent prospective clinical study involving eight patients who underwent ACL graft reconstruction, it was found that ^18^F-FDG PET could be performed at low radiation burden levels with sufficient image quality to evaluate graft healing [2].

Studies using ^18^F-FDG PET imaging after ACL reconstruction have demonstrated a significant increase in tendon graft metabolism during the immediate post-operative period, as well as during the first 2 years of post-operative healing [9, 20]. Recently, a study evaluated the utility of ^18^F-FDG PET /MRI in ligament imaging. This study followed 19 patients post-ACL reconstruction with autografts and allografts at four different time-points from 1.6 months up to 125.9 months [9]. The authors found that ^18^F-FDG PET/MRI could be used to evaluate metabolic activity of both types of ACL grafts. They also demonstrated that there was a statistically significant decrease in metabolic activity at 2 years post-operatively when compared to earlier time-points.

The purpose of this study was to utilize serial PET/MRI scans to evaluate graft metabolic activity and better understand the process of bio-integration of the ACL graft at precise time-points during the first post-operative year. We hypothesized that early on at the 3-month and 6-month scans there would be increasing biological activity as neovascularization occurs, which would downtrend at the 12-month scan during incorporation of the graft. The timeline of ligamento-osseus changes in the knee post-ACL reconstruction would be helpful in shaping safe return to play parameters in recreational/professional athletes.

## Methods

### Participants

Six patients (three males and females) were recruited into this institutional review board (IRB) approved prospective study from 2013 to 2016 (Table 1). This was designed as a pilot study to gain a better understanding of the time-points associated with graft healing and be balanced for sex. All patients underwent all-inside, dual suspensory fixation, quadrupled semitendinosus autograft hamstring ACL reconstruction by one sports fellowship trained orthopedic surgeon. A suspensory type cortical fixation device (ACL Tightrope® RT Arthrex, Naples, FL) was chosen both for its biomechanical characteristics and to allow for improved radiologic evaluation of graft integration within the tunnels without the presence of foreign body fixation. Tunnel widths ranged from 8 mm to 10 mm. Similar amounts of ACL stump were removed with no preservation attempts for all patients at both the femoral and tibial tunnel locations. All patients followed standardized post-operative rehabilitation protocols. Our protocol involves bracing, crutches, ROM exercises, and ice and elevation in the first post-operative week. In week 2, bracing is maintained, and patients weight bear as tolerated, ROM exercises continue, and strengthening is initiated. In week 3, bracing is discontinued and ROM and strengthening exercises are continued. Weeks 4–10 focus on progressive strengthening and neuromuscular control. Weeks 10–16 are the advanced activity phase and add sport-specific exercises. Finally, at 16–22 months patients are returned to all activities. The patients later underwent simultaneous ^18^F-FDG PET and MRI (Siemens Biograph mMR) of both the operative and non-operative knee (for control) at 3 months, 6 months, and 12 months post-operatively.

**Table 1:** Patient Demographics

Gender	Age	Height (cm)	Weight (kg)	BMI	Injury → Surgery (days)
	Mean StDev	Mean StDev	Mean StDev	Mean StDev	Mean StDev
**Male**	33.7 ± 9.5	173.7 ± 5.1	93.9 ± 17.3	31.2 ± 5.8	43.7 ± 9.0
**Female**	37.0 ± 11.3	161.7 ± 5.7	66.0 ± 11.5	25.1 ± 2.9	135.7 ± 147.2

The three male and female patients were compared based on age, height (centimeters), weight (kilograms), Body Mass Index (BMI), and time of injury to surgery (days)

Inclusion criteria for patients were primary unilateral ACL tear, skeletal maturity (age > 18 years), and patients undergoing all-inside, dual suspensory fixation, quadrupled semitendinosus autograft hamstring ACL reconstruction. Exclusion criteria were lateral collateral ligament (LCL), posterior cruciate ligament (PCL), or posterior lateral corner injury, greater than grade two medial collateral ligament (MCL) sprain, multitrauma, skeletal immaturity (age < 18 years), grade three Outerbridge classification changes in one or more compartments, revision case, prior surgery to either knee, ACL insufficient contralateral knee, prisoners, cognitively impaired adults, hospital employees, inability to undergo MRI secondary to non-compatible implantable devices, and females of child-bearing age with positive urine human chorionic gonadotropin (hCG). All patients meeting the criteria were offered inclusion into the study until the male and female groups were filled.

### Post-operative imaging

Patient preparation, radiopharmaceutical administration, image acquisition, and analysis followed standard clinical protocols as described below [20]. Patients were instructed to fast four-six hours prior to the intravenous administration of ^18^F-FDG to decrease physiologic glucose levels and to reduce serum insulin levels to near basal levels. Their blood glucose levels were checked via fingerstick before ^18^F-FDG administration. Since uptake of ^18^F-FDG is reduced in hyperglycemic states, the patients were only imaged if their blood glucose levels were less than 150 mg/dL. The patients remained recumbent for ^18^F-FDG administration and during the subsequent uptake phase to avoid muscular uptake. The recommended dose of ^18^F-FDG for an adult (70 kg) is 185–370 MBq (5–10 mCi), as an intravenous injection. For this study, we employed 185 MBq. PET scans were started at time of IV injection and were continued for 60 min. Only the last 20 min of the PET uptake data were used, which is equivalent to 40 min of uptake.

After PET acquisition, standard clinical MRI sequences, including sagittal and coronal proton density (PD), sagittal and coronal fat suppressed PD, axial fat suppressed T2 weighted, and additional 3D double echo ultrashort echo time (UTE) were acquired.

### Imaging analysis

**Fig. 1:**
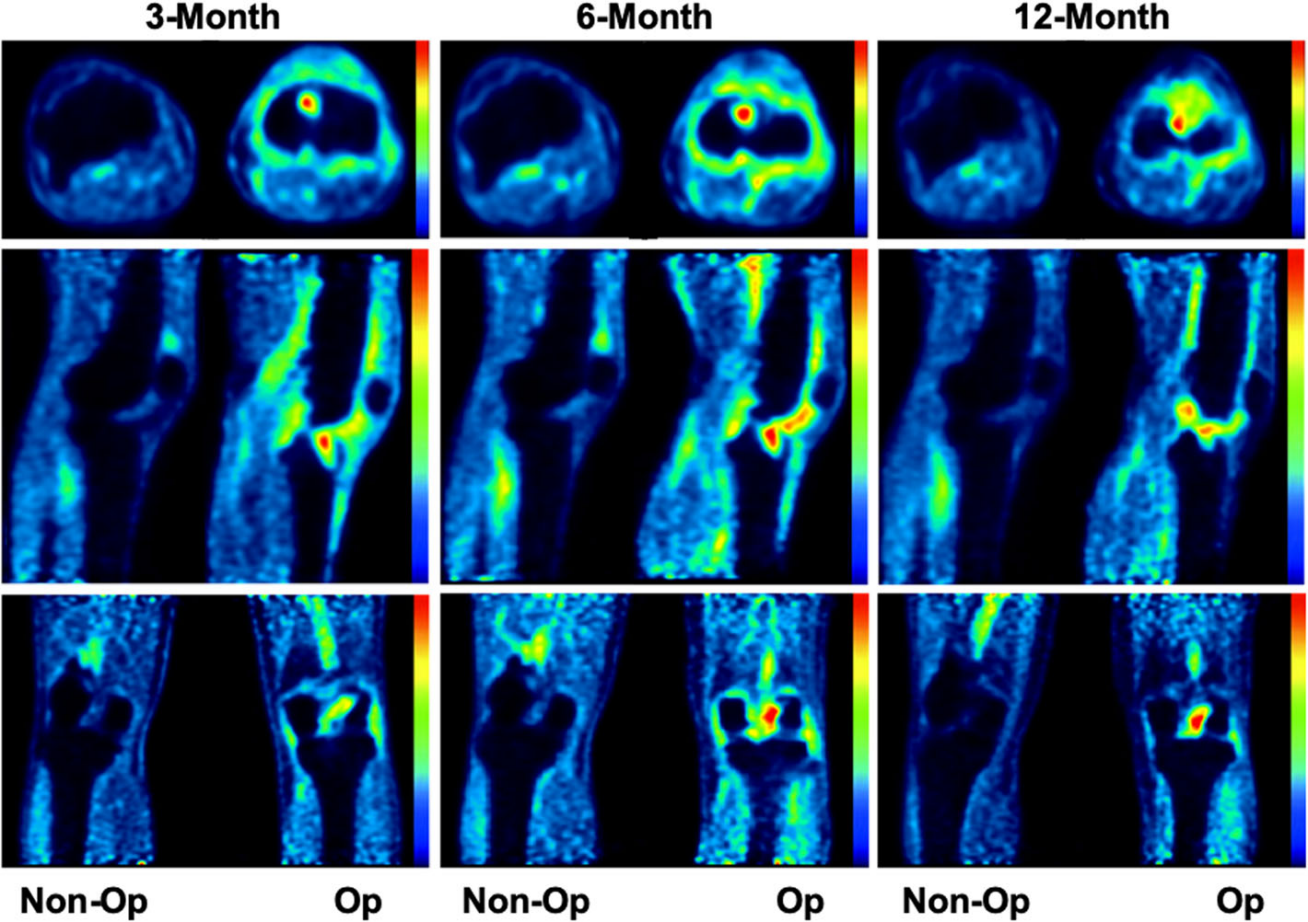
Time course of PET uptake following ACL reconstruction. Whole knee PET uptake regions of interest in non-operative (left) and operative (right) knees 3 months, 6 months, and 12 months post-operatively from a single representative patient. Images are taken from axial (top), sagittal (middle), and coronal (bottom) planes centered on the ACL. Increased ^18^F-FDG uptake is indicated by a shift from blue to red

**Fig. 2:**
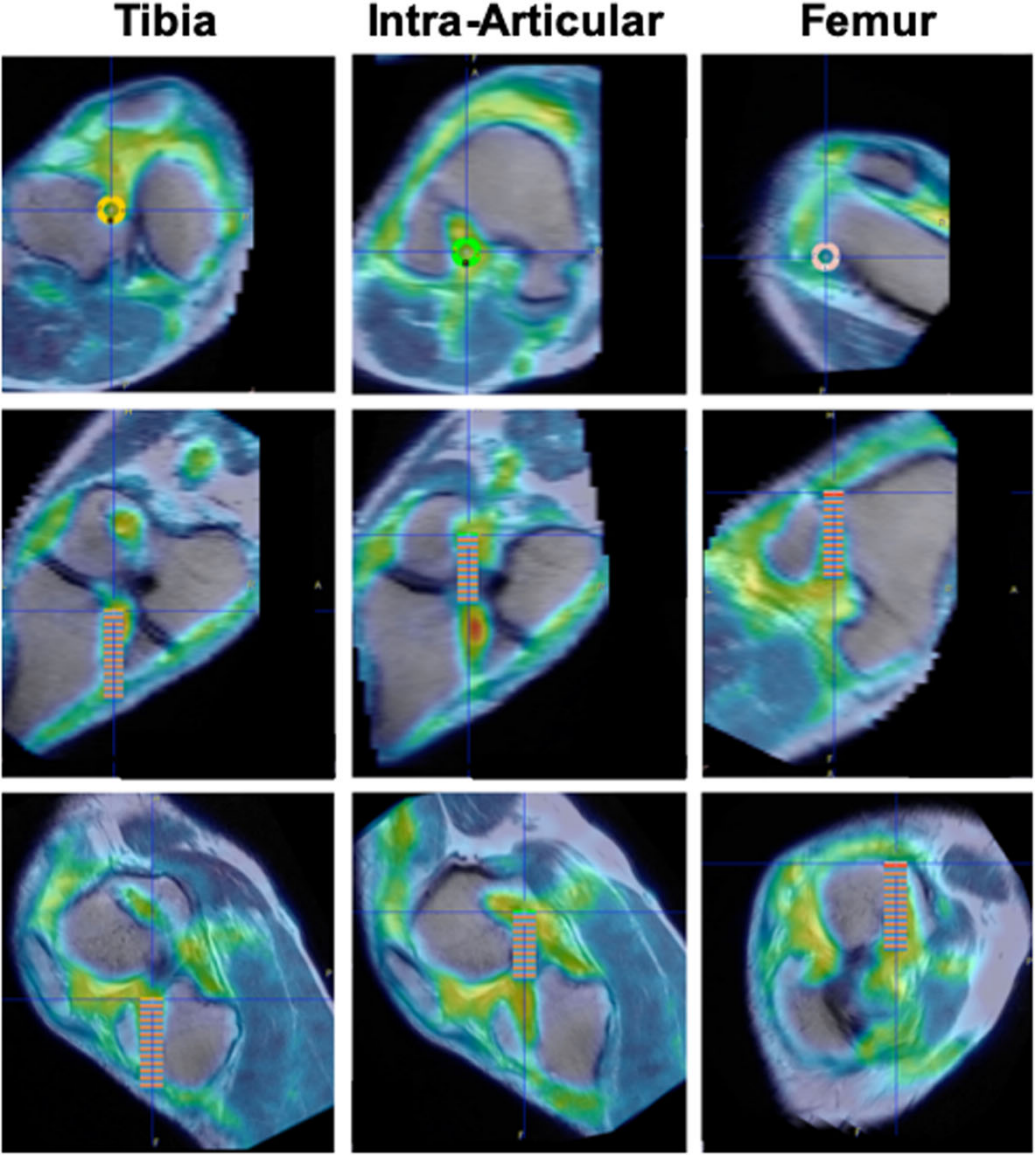
Realignment of PET/MRI images for quantitative analysis. Images showing the re-sliced PET/MRI images for the tibial tunnel, intra-articular graft, and femoral tunnel. The images were aligned so that the tunnels and graft were perpendicular to the imaging plane on axial (top), sagittal (middle), and coronal (bottom) views. Multiple circular regions of interest matching the tunnel diameters were then positioned on the images (colored overlay) to quantify regional ^18^F-FDG uptake

After image acquisition, whole-knee regions of interest (ROIs) were placed on both knees to characterize changes at the organ scale (Fig. 1). Then, for each tunnel (in both the femur and tibia), MRI images were digitally re-sliced to align the tunnels perpendicular to the image plane and circular ROIs were manually drawn on each plane with diameters equal to the tunnel diameters. The same re-slicing was applied to the corresponding PET images (Fig. 2). The standardized uptake value (SUV), which is a standard PET quantification method, was then calculated for each of these ROIs. The SUV normalized to body weight is given by the following equation: SUV = [AC_VOI_ (kBq/ml)]/[FDG dose (MBq)/BW (kg)], where AC_VOI_ is the attenuation-corrected mean radioactivity concentration value for the volume of interest and Bg is the blood glucose. Although no test-retest data is available specifically for ACL assessment, it’s accuracy is about 20% for tumor imaging when using the mean value over the region of interest, which is more accurate than using the maximum value [5].

In addition, sequential knee MRI images were qualitatively reviewed by a musculoskeletal fellowship trained radiologist for morphological changes or pathology.

### IRB approval

This study was approved by Stony Brook University’s IRB (CORIHS B) (FWA #00000125) as CORIHS#2014–2662-F.

### Statistical analysis

For statistical analysis, all of the patients were grouped together at each time-point and the data are reported as a group mean ± standard deviation. Total knee SUVs were compared between each patient’s operative and non-operative knee at each time-point using paired t-tests and differences were considered significant for values of *p* < 0.050. Total knee SUVs were averaged from all patients at each time-point and compared across time using Friedman’s Two-Way Analysis of Variance by ranks, with differences considered significant for values of *p* < 0.050. For the whole region and subregion SUV, the data were averaged from all patients at each time-point and compared across both region and time using Friedman’s Two-Way Analysis of Variance by ranks, with differences considered significant for values of *p* < 0.050. All tests were performed using SPSS Ver. 25 (IBM).

## Results

Total knee PET activity based on averaged SUV (Fig. 1, Table 2) was increased at all time points in the operative knee when compared to the non-operative knee (3-month, *p* = 0.000; 6-month, *p* = 0.001; 12-month, *p* = 0.006). The activity decreased significantly between the three-month and 12-month time-points in the operative knee (*p* = 0.022) with the activity at 1 year remaining 34% above that of the contralateral knee.

**Table 2:** Total Knee Standardized Uptake Value (SUV)

Time	Operative	Non-Operative	***p***-value
	Mean StDev	Mean StDev	
**3-Month**	0.6112 ± 0.1010	0.2906 ± 0.0617	0.0000^@^
**6-Month**	0.5587 ± 0.1338	0.3100 ± 0.0680	0.0010^@^
**12-Month**	0.4429 ± 0.0893	0.3332 ± 0.0698	0.0060^@^
***p*-value**	0.022^&^	0.165	

The operative knee was compared to the non-operative knee, and each knee within time-periods: three-month, six-month, and 12-month. ^&^ and ^@^ denote significance (*p* < 0.05) between the three-month and 12-month time-points and between the operative and non-operative knee, respectively

^&^3-Month vs. 12-Month, ^@^Operative vs. Non-Operative

**Fig. 3:**
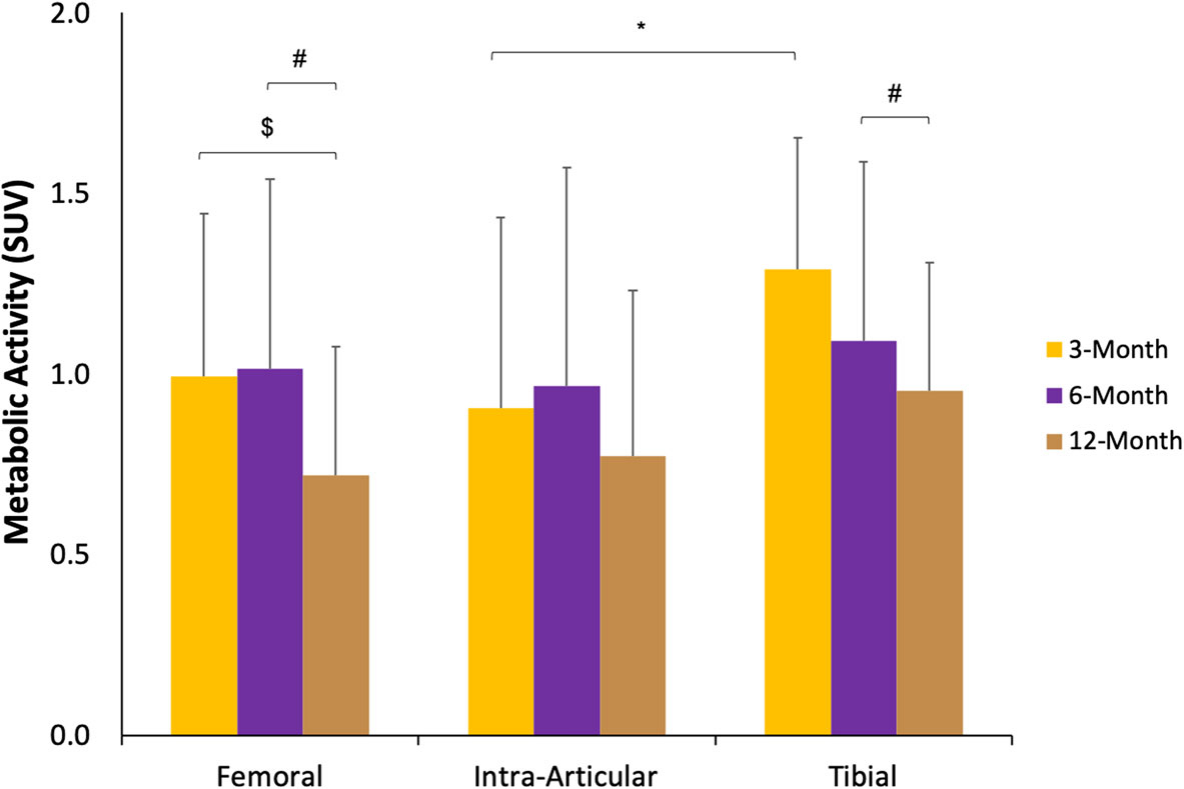
Whole Region SUV. Graph displaying the metabolic activity (y-axis) of the femoral tunnel, intra-articular graft, and tibial tunnel (x-axis). The metabolic activity of the whole regions is differentiated into the three-month (yellow), six-month (purple), and 12-month (brown) time periods. *, ^$^, and ^#^ denote significance (*p* < 0.05) between the tibial tunnel and intra-articular graft, three-month and 12-month time-periods, and 6-month and 12-month time-periods, respectively

To evaluate the knee at organ-scale, localization was initialized broadly, then advanced to be increasingly specific. When the knees were separated into three distinct whole regions (tibial tunnel, intra-articular graft, femoral tunnel) [Fig. 3], there was a statistically significant difference at the three-month time-point, with increased PET activity in the tibia when compared with the activity within the tibial and intra-articular regions (*p* = 0.006). When comparing the regions as a function of time, the tibial region had more activity at the six-month time-point than at the 12-month (*p* = 0.017), while the femoral region had more activity at three- and six-month time-points than at the 12-month (*p* = 0.000). No differences were seen when comparing the femoral regions as a function of time.

**Fig. 4:**
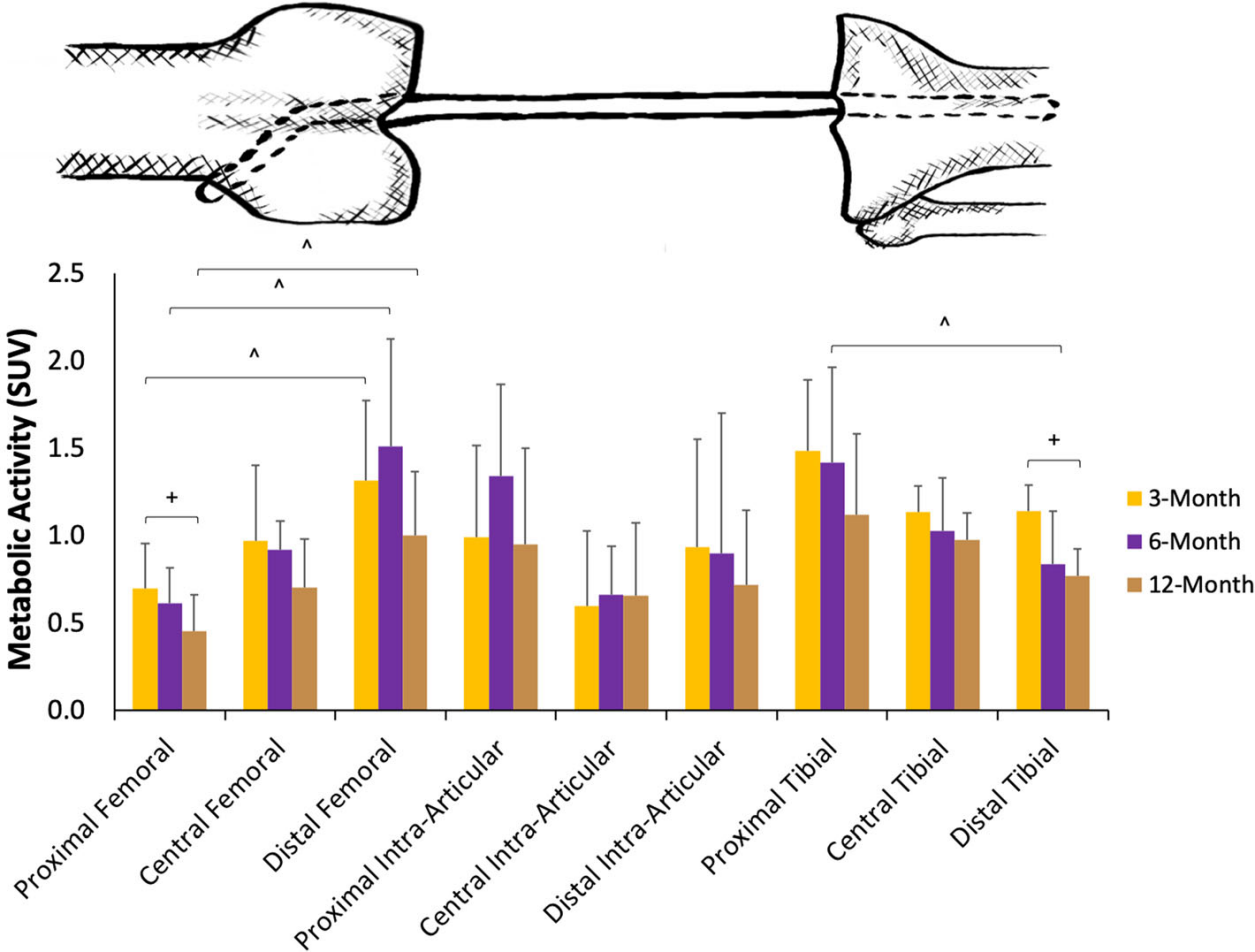
Subregion SUV. Graph displaying the metabolic activity (y-axis) of the femoral tunnel, intra-articular graft, and tibial tunnel split into proximal, central, and distal subregions (x-axis). An exaggerated sketch of the subregions is overlaid above to correlate with the subregions on the x-axis. The metabolic activity of the subregions is differentiated into the three-month (yellow), six-month (purple), and 12-month (brown) time periods. ^ and ^+^ denote significance (*p* < 0.05) between the distal and proximal subregions and the six-month and 12-month time-periods, respectively

**Fig. 5:**
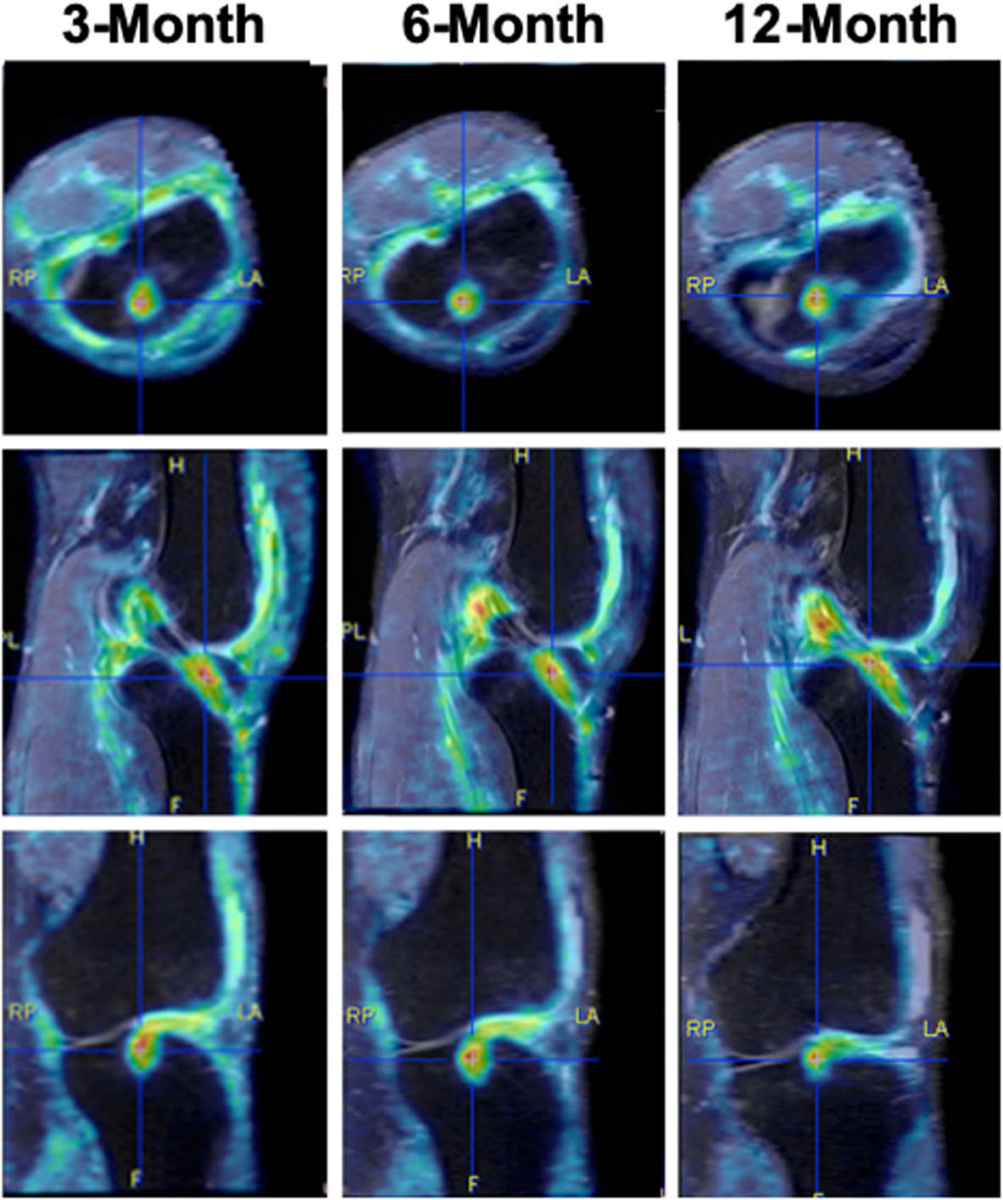
PET imaging shows focal areas of increased uptake. This figure shows three-month, six-month, and 12-month post-operative PET/MRI scans from the same patient scaled to same standardized uptake value (SUV) in the axial (top), sagittal (middle), and coronal (bottom) planes. Focal areas of high ^18^F-FDG uptake (red) are apparent, as well as an overall decrease in uptake over time

When the tunnels and graft were divided into sub-regions (distal, middle and proximal 1/3 of the tibial tunnel, intra-articular graft, and femoral tunnel) [Fig. 4], the tibial tunnel had more activity distally than proximally (*p* = 0.015) at the six-month time-point. Within the tibial sub-regions, there was more activity in the distal sub-region at the six-month time-point than at the 12-month (*p* = 0.015). There were no significant differences within the intra-articular graft sub-regions by location or time. Within the femoral tunnel sub-regions, there was more activity distal than proximal at three- (*p* = 0.006), six- (*p* = 0.007), and 12-month (*p* = 0.006) time-points. Proximally, there was more activity at the three-month than at 12-month time-point (*p* = 0.022). Overall, when observing the activity of the sub-regions, there appeared to more ^18^F-FDG activity at the surface of the joint. Focal areas of activity were also noted within certain areas of both tunnels and within the intra-articular graft of all patients (Fig. 5).

## Discussion

The most important finding in this study is that even a year post-ACL reconstruction, the process of ligamentization of the tendon graft is ongoing compared to the contralateral non-operative knee. The ligamentization and neovascularization process has been well studied in both human and animal models with histologic and advanced imaging protocols [1, 3, 4, 6, 8, 11, 16, 21, 24]. In addition, the biomechanical properties of healing graft tissue have been studied and demonstrated modes of failure that correlate with specific times and remodeling activities [3, 22]. This study adds to the growing literature supporting ^18^F-FDG PET/MRI as an imaging modality to assess vascularization and incorporation of the graft in ACL reconstruction. With considerable variation between patients, it was important to normalize values to the patient’s own contralateral knee.

Post-operative ^18^F-FDG PET/MRI evaluations of ACL hamstring graft reconstructions clearly demonstrate evolving biologic activity within the intra-articular region of the graft, as well as the femoral and tibial tunnel fixation points over time. The quantitative decrease in ^18^F-FDG uptake over time for both the whole knee, the tunnels, and graft confirmed our hypothesis that as the incorporation process occurs there would be a decrease in uptake within the knee. Although this activity decreases over time, considerable generalized activity remains, even at 1 year. This finding points to an ongoing incorporation process that has not resolved at the 1-year time-point. Prior studies have observed distinct morphologic changes within the graft after 1 year and suggested that changes may continue in the graft up to 2 years post-surgery [1, 16, 18, 21]. Continued elevation in generalized PET activity indicates that there is additional biologic information regarding the post-operative reconstructed knee than traditional studies have shown by either MRI or histology.

Of note, there was considerable variation in whole knee ^18^F-FDG uptake was detected from patient to patient. Our scanning protocol attempted to control for this, and therefore the variability is attributed to each patients’ individual biologic response to surgery. This observation supports our study design to obtain a scan of the normal contralateral knee at each time-point for comparison purposes.

Focal areas of increased and decreased ^18^F-FDG PET activity were identified within the tibial and femoral tunnels over time (Fig. 5), however there was not enough consistency between samples to allow for quantitative analysis of this phenomenon. Increased PET activity was encountered in both tunnels, particularly near the joint surfaces, and may be indicative of ligamento-osseous morphologic changes. We initially hypothesized that the graft would become increasingly biologically active over time as neovascularization occurs; however, this does not appear to hold true.

### Limitations

Limitations of the study include the small number of patients enrolled. As well, each of the patients elected to participate in this study, creating a potential selection bias. Also, one patient did not complete the scan at the 6-month time-point. The patients were grouped together for analysis to evaluate the effects of the study in a diverse population. Thus, future studies will be powered to identify differences in outcomes between sexes. Given that there was PET activity remaining on the scans at 1 year, those future studies would also benefit from including scans out to 2 years to evaluate for normalization. Comparison of these PET data to SPECT data would also be of benefit. Perhaps more importantly, future studies to correlate PET scans to biomechanical strength will be vital for evaluating return to play for athletes, as well as return to daily activities. PET scans performed with ^18^F-FDG are known to detect the metabolic activity of inflammatory cells and because of this, some enhanced activity that is seen may be secondary to inflammation versus actual neovascularization. However, one prior study demonstrated that there are no inflammatory cells 3 and 8 weeks post-operatively on second look arthroscopic biopsies of ACL grafts. Another demonstrated no signs of inflammation 7 weeks post-operatively on histologic evaluation of a whole knee specimen. Inherently, while the effect of inflammation in our study is unknown, it is assumed to be marginal [8, 11]. Specific targeting radiopharmaceuticals could be considered to address this issue. Finally, there also is some inherent lack of spatial resolution associated with the acquisition of the PET scan data. Utilizing a higher resolution detector could aid in improving the spatial resolution although it is not known if this would change the results or have any additional significance.

## Conclusion

PET/MRI evaluation of ACL graft reconstructions demonstrate evolving biologic activity within the graft and both tunnels. Focal areas of increased activity within the tunnels may be indicative of ligamento-osseous morphologic changes. The operative knee continues to have considerable activity at 1 year out from surgery especially when compared to the contralateral non-operative knee. This suggests ongoing bio-integration of the graft beyond the first post-operative year. With clinical correlation of patient symptoms, measurement of biologic activity within the graft with ^18^F-FDG PET/MRI can lead to a more detail return to function/play protocol.

## Data Availability

All data generated or analyzed during this study are included in this published article.

## Abbreviations

ACL: Anterior Cruciate Ligament
MRI: Magnetic Resonance Imaging
PET: Positron Emission Tomography
ACLT: Anterior Cruciate Ligament Transection IRB Institutional Review Board LCL Lateral Collateral Ligament PCL Posterior Cruciate Ligament MCL Medial Collateral Ligament
HCG: Human Chorionic Gonadotropin
SUV: Standardized Uptake Value AC_VOI_ Attenuation-corrected mean radioactivity concentration in a volume-of-interest
BW: Body Weight
PD: Proton Density
UTE: Ultrashort Echo Time
